# Translational interdisciplinary research on human chorionic gonadotropin’s enhancement of neuroendocrine crosstalk: a novel medical hypothesis for systemic adjunctive treatment of psychiatric disorders

**DOI:** 10.3389/fpsyt.2025.1537442

**Published:** 2025-04-29

**Authors:** João Francisco Pollo Gaspary, Luis Felipe Dias Lopes, Antonio Geraldo Camara

**Affiliations:** ^1^ Instituto AuBento – Center for Teaching, Clinical Practice and Research in Orthomolecular and Translational Health Innovation, Santa Maria, Brazil; ^2^ Federal University of Santa Maria, Santa Maria, Brazil; ^3^ Center for Social and Human Sciences, Postgraduate Program in Administration, Federal University of Santa Maria, Santa Maria, Brazil; ^4^ Institute Camara – Center for Clinical and Orthomolecular Practice, Ribeirão Preto, Brazil

**Keywords:** psychiatric disorders, hormetic therapy, neuroplasticity, systemic inflammation, insulin resistance, sex hormone production, hypothalamic activity, personalized treatment

## Abstract

**Introduction:**

It is increasingly recognized that the brain continuously interacts with other body systems such as the immune system, the gut-brain axis, and the endocrine system. Dysfunctions in these systems can impact mental health by altering neurotransmitter levels and the neurochemical environment. This shift in understanding underscores the need for therapeutic strategies that address systemic health and mitochondrial function, alongside psychosocial aspects of the disease, offering a more personalized and adaptive approach to treatment.

**Methodology:**

This study utilizes a translational research approach structured through the Work Breakdown Structure methodology, dividing the process into six interconnected Work Packages (WPs). These include systematic literature reviews on endocrine dysfunctions and hormonal therapies in mental disorders, application of Design Thinking for neuroendocrine innovation, and hypothesis exploration of hCG as a systemic adjunctive treatment for psychiatric disorders, culminating in result dissemination and evaluation.

**Results:**

Work The study identified multiple mechanistic impacts of human chorionic gonadotropin (hCG) relevant to psychiatric treatment. Key findings from hCG Hormetic Therapy (HHT) include stimulation of sex hormone production, reduction of insulin resistance and systemic inflammation, enhancement of hypothalamic activity to regulate appetite, sleep, and emotions, and LH-like effects on cognition. HHT also increases IGF-1 availability, promoting neuroprotection, cognitive improvements, and reduced mitochondrial dysfunction, restoring cellular function critical for brain health.

**Implications for Clinical Practice:**

The findings underscore the significance of enhancing endocrine and metabolic functions as a viable strategy for improving psychiatric care, aligning with trends that advocate holistic treatment strategies. The suggested dose for future research protocols is 500 IU IM per week for at least 10 weeks.

**Conclusion:**

Supporting diverse and varied research is crucial for advancing medical knowledge. Continuous exploration of neuroendocrine dysfunctions in mental disorders using advanced tools from neuroscience, endocrinology, and psychiatry can provide new pathways for more effective and personalized treatments. The study of HHT effects offers insights into complex neuroendocrine interactions, underscoring the potential for innovative therapeutic strategies in psychiatry.

## 1 Introduction

The paradigm of mental disorders has undergone a significant transformation, moving beyond the traditional neurotransmitter imbalance hypothesis to embrace a systemic perspective that includes inflammatory processes (1–3) and mitochondrial dysfunction (4–7). This shift highlights the complexity of psychiatric conditions and underscores the intricate interplay among various bodily systems, such as the endocrine, immune, and neurological networks (8–10), emphasizing that mental health is the result of overall bodily health (11).

While current psychiatric guidelines, such as those outlined by Kennedy et al. (12) and Lam et al (13) for major depression and Yatham et al. (14) for bipolar disorder, provide comprehensive protocols based on the best available evidence, there is a notable reliance on treatments supported by Level 2 evidence. This scenario not only highlights the challenges of gathering robust clinical data but also points to the potential limitations of conventional treatment modalities that primarily target neurotransmitter systems. The prevalence of Level 2 evidence for most psychological interventions reflects a broader issue within psychiatric research. Specifically, it underscores the difficulty of conducting randomized controlled trials in the mental health field due to ethical considerations, variability in patient responses, and the subjective nature of psychological outcomes. Moreover, the insufficient evidence for several therapies suggests a significant gap in our understanding and underscores the need for more rigorous and extensive research.

These apparent gaps in the guidelines highlight the necessity for integrative treatment approaches that address more complex biological interactions, such as those influenced by mitochondrial dysfunctions and neuroendocrine factors. Furthermore, this situation underlines the importance of considering innovative approaches that may not yet be thoroughly explored or understood within the conventional frameworks used in current psychiatric guidelines (15). This discussion integrates a critique of current guidelines and emphasizes the need for a broader and more dynamic approach to research and treatment in psychiatry, aligning with the innovative aspects of how and what to treat. MacEwan et al. (16) highlights the necessity of stimulating public and private investment in the research and development of novel and effective treatments and approaches for mental disorders.

This emergent divergence in understanding mental disorders—from viewing them as results of isolated neurotransmitter imbalances to manifestations of systemic dysfunctions—reflects the complexity of human psychopathology and underscores the need for a holistic and integrative approach in diagnosis and treatment. Recognizing the importance of interactions among various bodily systems and brain function can lead to significant advances in how we understand and treat mental disorders (1, 17, 18).

In addition to the evolving paradigms within psychiatric treatment, ongoing discussions in the schizophrenia research community underscore the importance of addressing broader cognitive impairments and their systemic biological bases (19–22). Recent research into neuroinflammation has highlighted its potential role in the cognitive deficits seen in schizophrenia, which are often overshadowed by the focus on specific neural targets. These recent studies into neuroinflammation have highlighted its potential role in the cognitive deficits seen in schizophrenia, which are often overshadowed by the focus on specific neural targets. These studies suggest that these deficits may stem from widespread neural network dysfunctions, including abnormalities in neurotransmitter systems like glutamate and gamma-aminobutyric acid, and potentially extending to broader systemic issues such as energy metabolism and inflammatory processes. Such understandings can also be extrapolated to other mental disorders (23, 24).

Building on this perspective, our study focuses on the systemic interactions within psychiatric disorders, particularly neuroendocrine crosstalk and mitochondrial dysfunction, beginning with the objective of exploring the role of endocrine dysregulation in mental health, seeking to obtain a comprehensive theoretical framework. This analysis is critical because endocrine imbalances, such as those involving thyroid, adrenal, and sex hormones, can profoundly affect brain function and contribute to the pathophysiology of psychiatric disorders. Moreover, these imbalances can be one of the initial triggers for mitochondrial dysfunction, further exacerbating neuropsychiatric symptoms.

Subsequently, the study seeks to evaluate the potential therapeutic benefits of hormonal interventions as adjunctive treatment for mental disorders. Given the systemic nature of hormonal influences on the body and brain, the objective is to understand and identify how hormonal therapies can be optimized to improve mental health outcomes. By analyzing the effects of these therapies, we can better understand their potential to mitigate systemic dysfunctions that underlie psychiatric conditions.

This integration of mitochondrial activation, energy metabolism, and inflammatory processes emphasizes the connection between neuroendocrine crosstalk and psychiatric diseases, reinforcing the relevance of our study’s approach in addressing these complex interactions. It situates our research within a broader context of emerging evidence that supports a systemic view of psychiatric disorders, highlighting the innovative potential of targeting mitochondrial pathways alongside neuroendocrine mechanisms.

In this context, this study aims to bridge neuroendocrine crosstalk and contemporary science, investigating whether specific actions can be validated through scientific rigor and exploring how these stimuli could be integrated into modern medicine for public health benefits. By identifying the biological effects that could be obtained from these stimuli, we aim to propose innovative therapeutic alternatives. To achieve this, design thinking and open innovation will be applied to suggest therapeutic alternatives that could eventually be considered Level 1 evidence.

## 2 Methods

**Figure 1:**
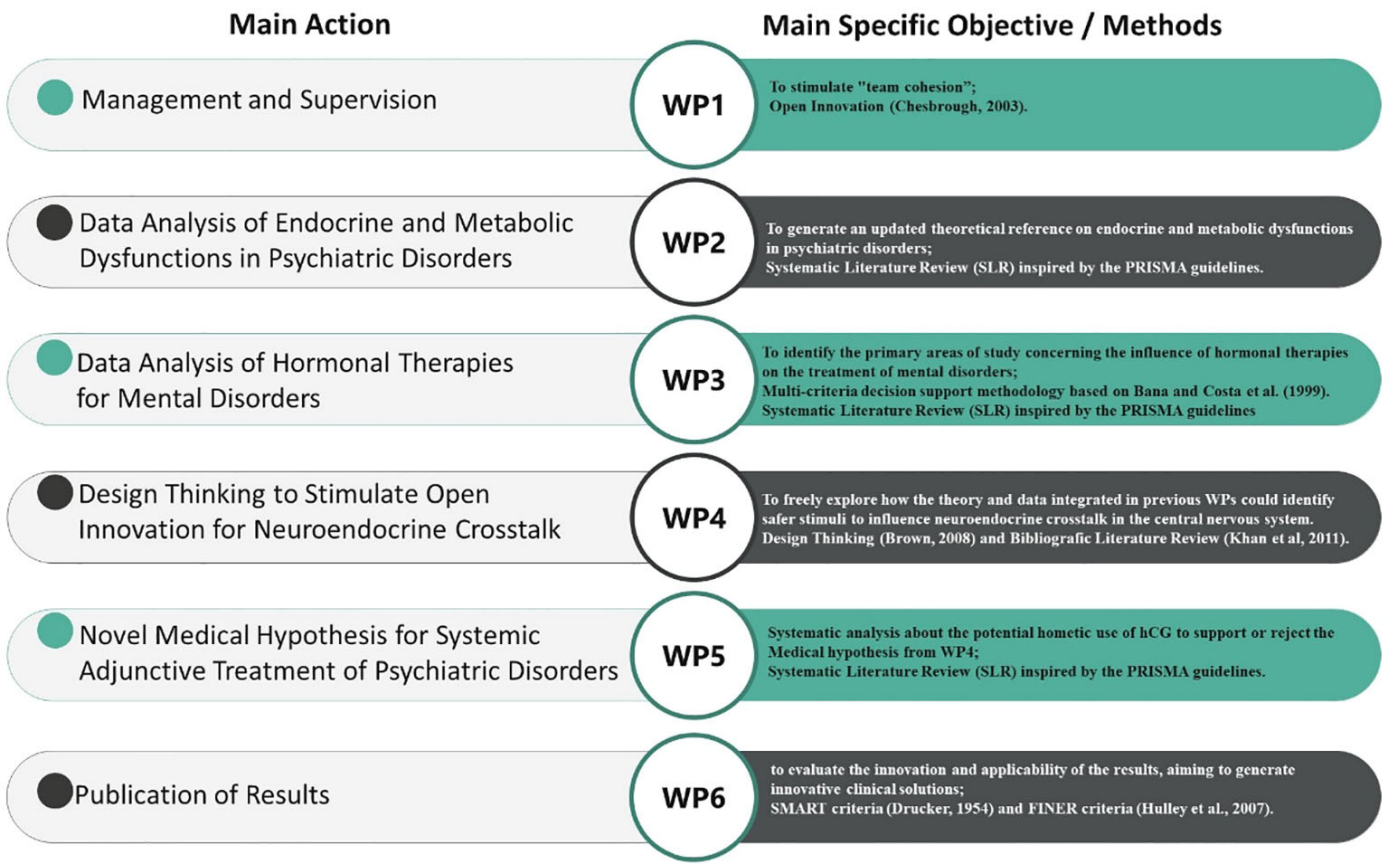
WBS methodology applied in this research.

To achieve the proposed objectives, Translational Research was adopted, structured through the Work Breakdown Structure (WBS) methodology as described by the Project Management Institute (PMI) (25, 26). This management approach divided the research process into six smaller, well-defined, interconnected Work Packages (WPs), each designed to explore specific aspects of Neuroendocrine Crosstalk in the CNS and mental disorders, promoting a comprehensive and multidisciplinary perspective (**Figure 1**). Each WP was followed by a detailed qualitative analysis of the results to ensure the relevance and applicability of the findings in a clinical context.

### 2.1 WP1

**Figure 2:**
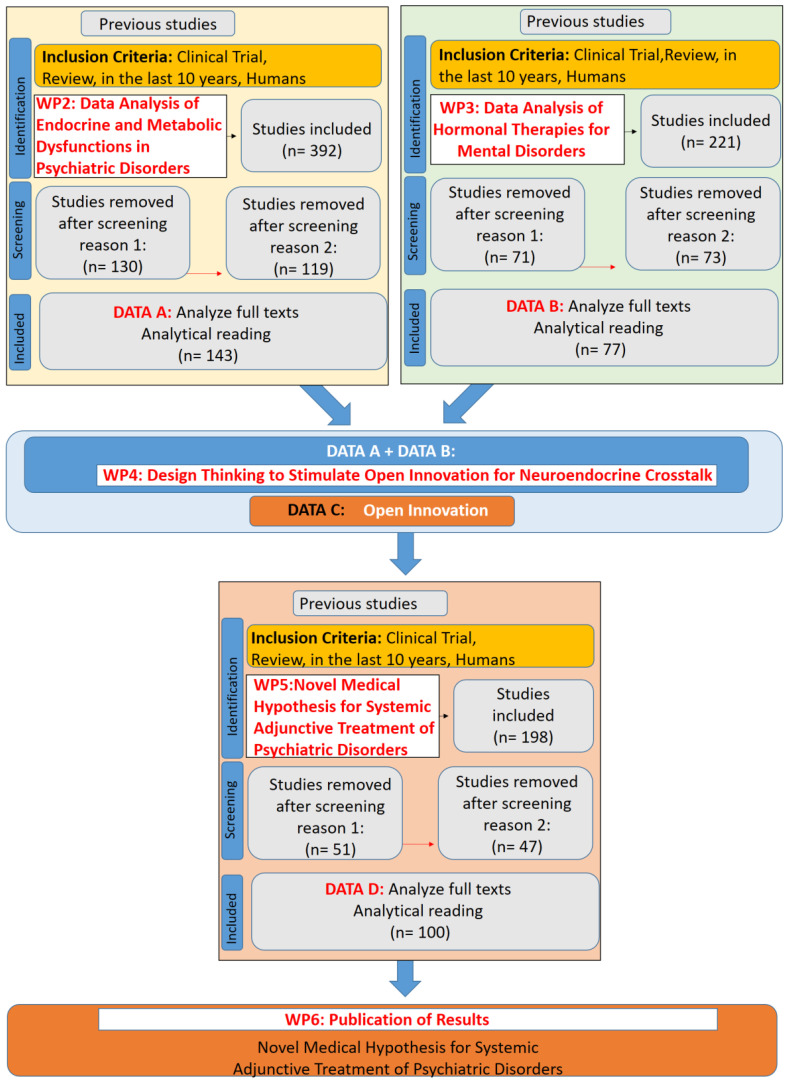
PRISMA-Compliant Flowchart Integrating Work Breakdown Structure (WBS) Methodology and Systematic Reviews for the Development of a Novel Medical Hypothesis.

The specific objective of WP1 (Management and Supervision) was to ensure the correct functioning of the project and its execution based on the initially outlined objectives and timeline, to stimulate “team cohesion” using the methodology of Open Innovation, to review and adjust each WBS as the project progressed, to ensure clear mechanisms for feedback and communication among the WPs, and to assess the impact of newly developed medical hypotheses. The methodological flow of this study, integrating the WBS framework with systematic reviews, is formally structured in **Figure 2**, which presents a PRISMA-compliant flowchart (27) detailing the inclusion and exclusion of studies at each stage, ensuring transparency and reproducibility in the research process. Additionally, a specific flowchart for each stage (WP2, WP3, and WP5) will be presented separately to provide a detailed breakdown of the screening and selection process in each phase.

### 2.2 WP2

The specific objective of WP2 (Data Analysis of Endocrine and Metabolic Dysfunctions in Psychiatric Disorders) was to generate an updated theoretical reference on endocrine and metabolic dysfunctions in psychiatric disorders. To achieve this, a Systematic Literature Review (SLR) was conducted, gathering information using the following eligibility criteria: descriptors in English: “Inflammation” and “mental disorders” and “psychiatric disorders” (resulting in 138 studies); “hypothalamic dysfunction” and “depression or anxiety or mental disorders” (resulting in 28 studies); “mental disorders” and “endocrine dysfunction or imbalance” (resulting in 169 studies); “mental disorders” and “psychiatric disorders” and “metabolic comorbidity” (resulting in 38 studies); and “psychoneuroimmunology” and “mental disorders” (resulting in 19 studies). This resulted in a total dataset of 392 studies.

**Figure 3:**
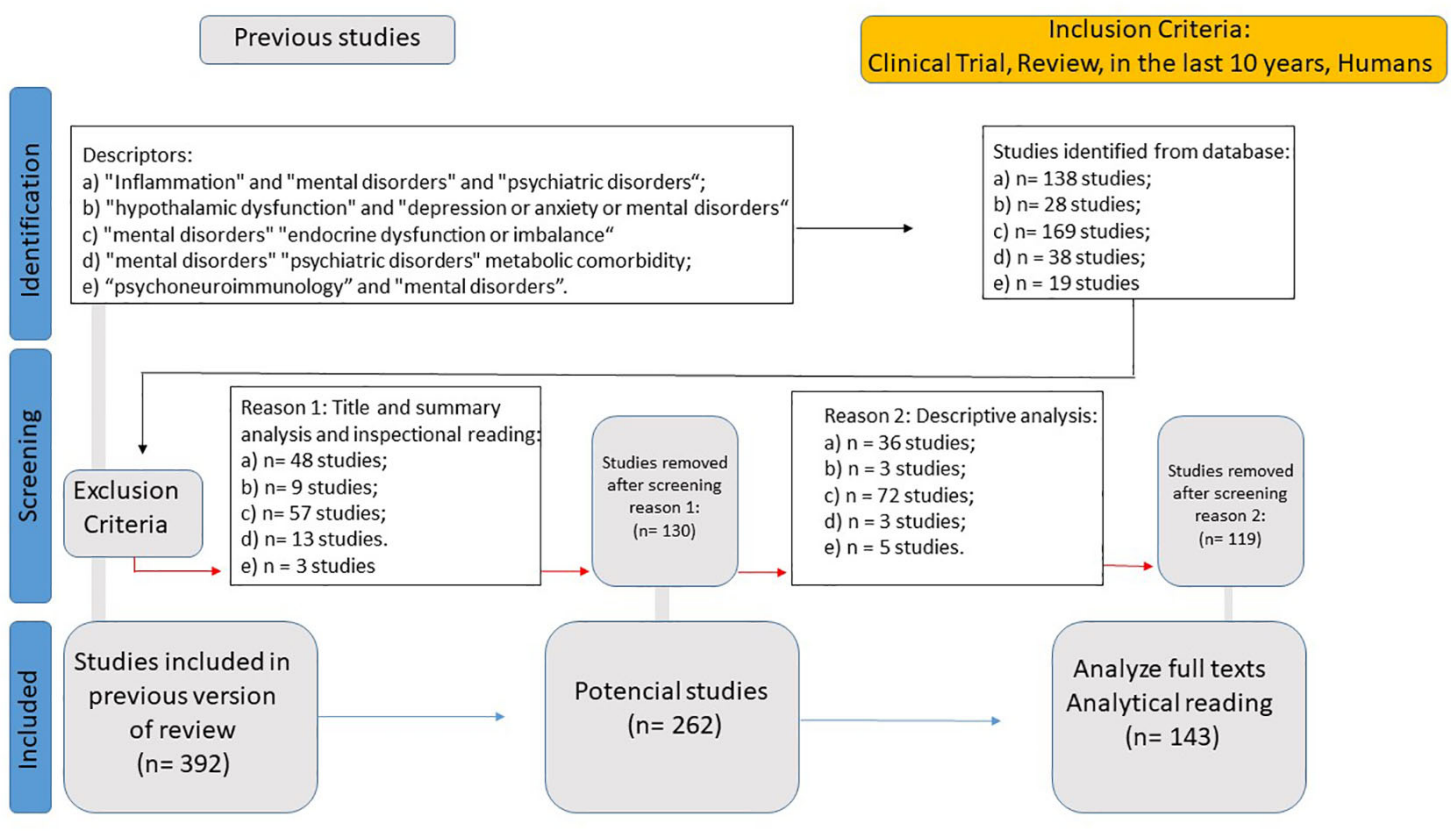
Systematic literature review flowchart for endocrine and metabolic dysfunctions in psychiatric disorders.

Exclusion criteria included studies that lacked a direct association with psychiatric disorders and endocrine/metabolic dysfunctions, articles focusing solely on animal models without translational applicability, and research that did not present relevant clinical outcomes or biochemical mechanisms related to neuroendocrine dysfunctions. After selection, the process yielded 143 articles for full analysis (**Figure 3**).

### 2.3 WP3

The specific objective of WP3 (Data Analysis of Hormonal Therapies for Mental Disorders) was to identify the primary areas of study concerning the influence of hormonal therapies on the treatment of mental disorders, generating a selection of Fundamental Points of View (FPVs) according to a multi-criteria decision support methodology based on Bana e Costa et al. (28). To this end, a new Systematic Literature Review (SLR) was conducted, gathering data using the following eligibility criteria: Clinical Trial, Review, in the last 10 years, Humans, and descriptors in English: “hormonal therapy” “mental disorder” “psychiatric disorder” (resulting in 0 studies); “hormone replacement therapy” “mental disorder” “psychiatric disorder” (resulting in 0 studies); “Testosterone replacement therapy” “mental disorder” (resulting in 0 studies); “testosterone replacement therapy” mood (resulting in 35 studies); “hormone replacement therapy” mood (resulting in 186 studies).

**Figure 4:**
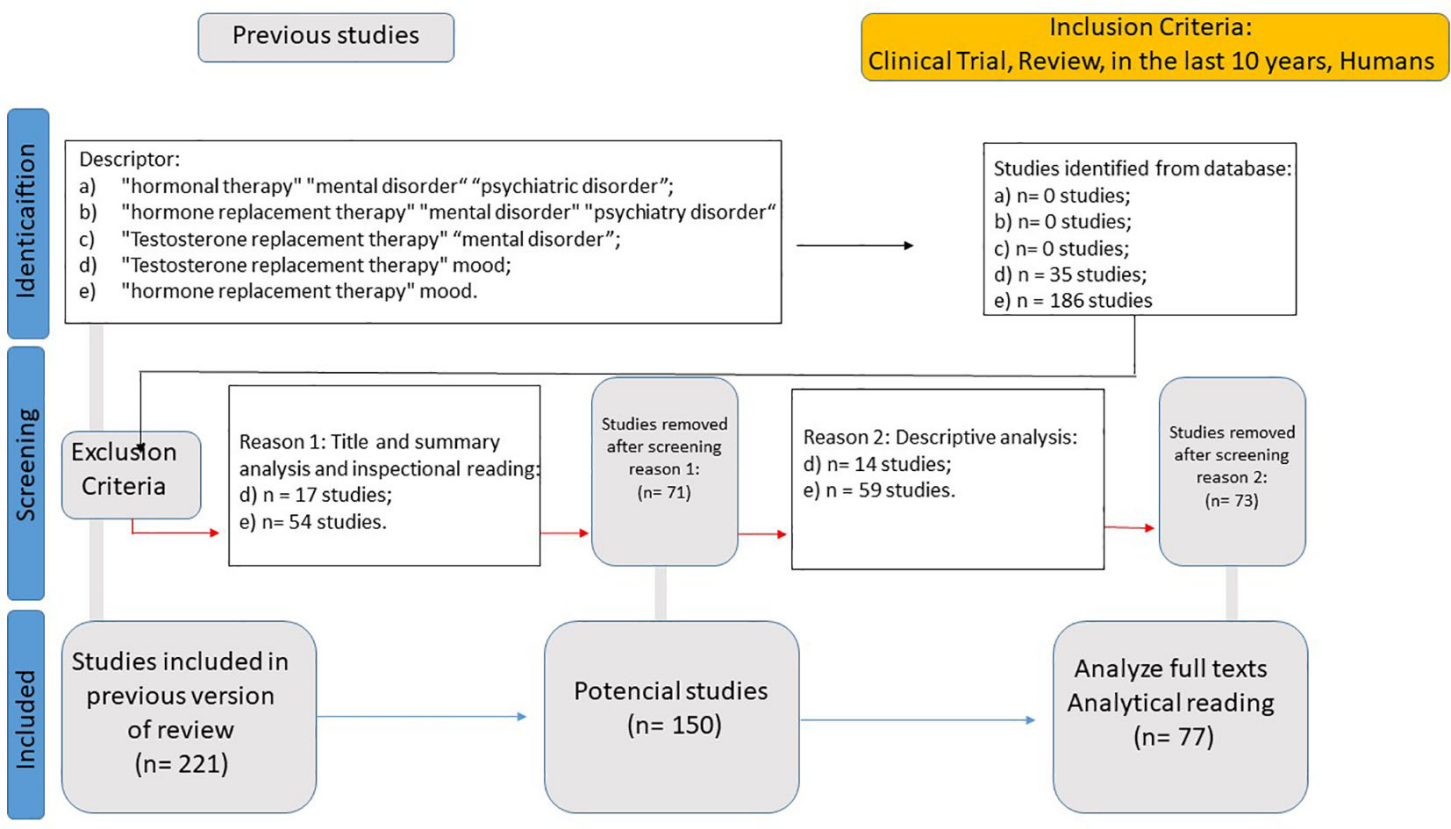
Systematic literature review flowchart for hormonal therapies in psychiatric disorders.

Exclusion criteria included studies that focused on hormonal replacement therapies without direct evaluation of their psychiatric or neurobehavioral effects, research exclusively based on animal models with no translational relevance, and articles that lacked methodological rigor in assessing clinical outcomes related to hormonal modulation in mental disorders. This resulted in a total dataset of 221 studies. After selection, this process yielded 77 articles for full analysis (**Figure 4**).

### 2.4 WP4

The specific objective of WP4 (Design Thinking to Stimulate Open Innovation for Neuroendocrine Crosstalk) was to freely explore how the theory and data integrated in previous WPs could identify safer stimuli to influence neuroendocrine crosstalk in the central nervous system, utilizing Design Thinking (29) and Open Innovation (30) to connect new theoretical propositions with practical applications. The choice of these methodologies underscores a human-centered approach to identifying innovative and creative solutions that address complex problems within neuroendocrine crosstalk. Through Design Thinking, the existence of an iterative process involving empathy, definition, ideation, prototyping, and testing is implied. Moreover, the Open Innovation methodology emphasizes the intent to collaborate beyond the traditional boundaries of the organization, involving a broader community of researchers, clinicians, and possibly even patients in the innovation process. This can facilitate the sharing of ideas, resources, and technologies to accelerate the development of new therapies and interventions. For idea testing, a broad-range literature review based on Khan et al. (31) was conducted freely and simultaneously until the idea was accepted by the researchers. The intention was to generate an ideation process constantly checked against the available scientific literature; hence, the inclusion criteria for analysis were broad, with the aim of ideas testing. The selection criteria are listed in **Table 1**.

**Table 1:** Article selection criteria in a review for this study based on Khan et al. (31) for WP4.

Criteria	Variables
Database	LILACS; Medline; Web of Science; Scopus; SciELO; Google Scholar; Research Gate; ClinicalTrials.gov; Patentscope; Prospero
Timeframe	All studies published until 2024
Languages	English, Portuguese, and Spanish
Indexed terms	Descriptors in English were generated from the iterative process.
Inclusion criteria for analysis	Broad, with the aim of idea testing

### 2.5 WP5

The specific objective of WP5 (Exploring a Novel Medical Hypothesis: hCG as a Systemic Adjunctive Treatment for Psychiatric Disorders) was to employ a systematic review about Hcg with the intention of obtaining direct or indirect data to support or reject the proposal resulting from WP4. For this, a Systematic Literature Review (SLR) was conducted, gathering data using the following eligibility criteria: Clinical Trial, Review, in the last 10 years, Humans, and descriptors in English: “hCG” “biological functions or clinical applications”.

**Figure 5:**
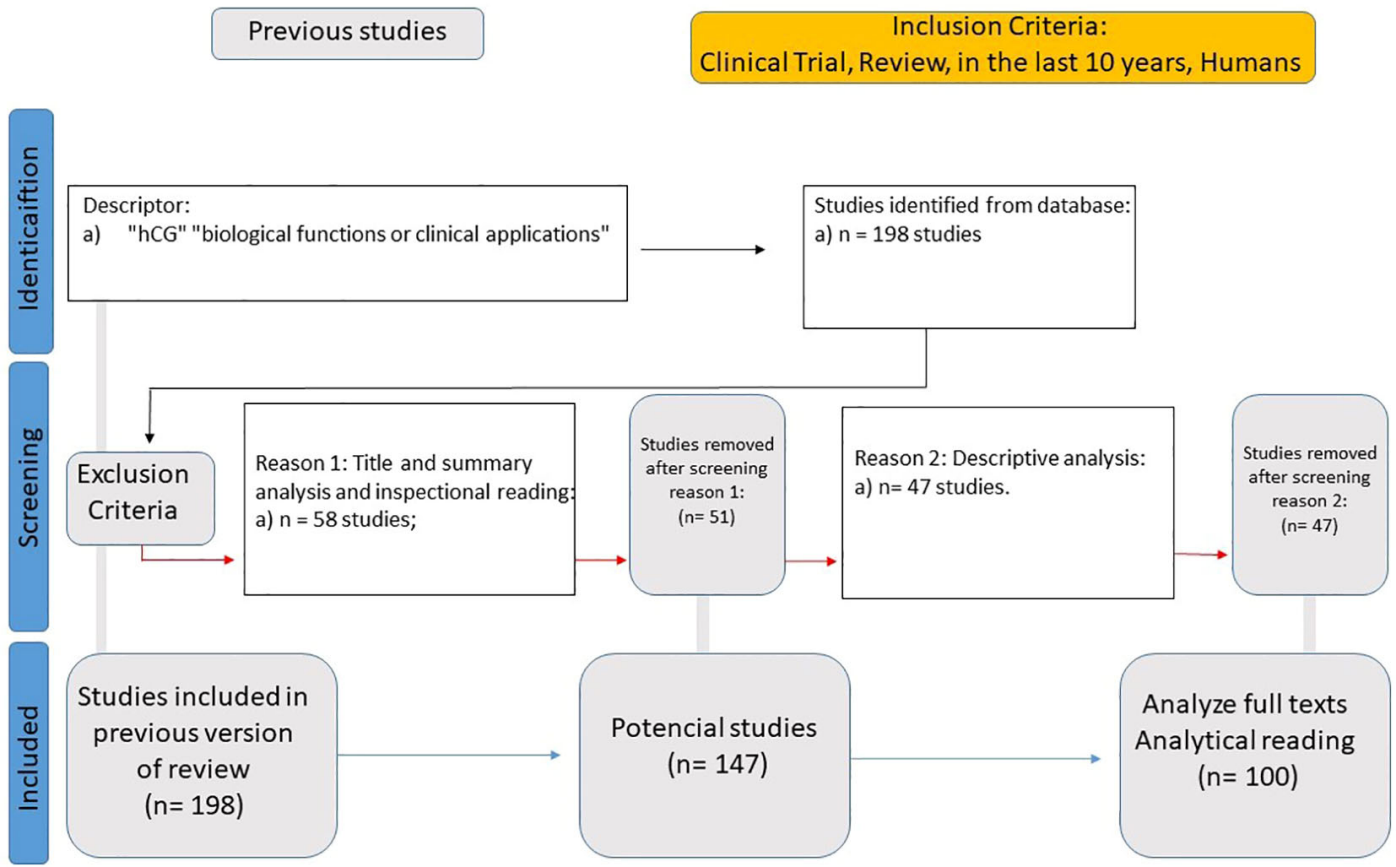
Systematic literature review flowchart for human Chorionic Gonadotropin (hCG) as an adjunctive treatment for psychiatric disorders.

Exclusion criteria included studies that focused on hCG applications unrelated to neuroendocrine or psychiatric functions, research solely on fertility treatments, oncological markers, or pregnancy-related outcomes without relevance to mental health, and preclinical studies without direct translational implications for psychiatric disorders. Additionally, studies with methodological limitations, such as inadequate sample sizes or lack of control groups, were excluded to ensure the reliability of the systematic review’s findings. This resulted in a total dataset of 198 studies. After selection, this process yielded 100 articles for full analysis (**Figure 5**).

### 2.6 WP6

The specific objective of WP6 (Publication of Results) is to evaluate the innovation and applicability of the results using SMART criteria (32) and FINER criteria (33), aiming to generate innovative clinical solutions. Furthermore, we will discuss the methodological limitations and ethical considerations involved in the research, especially concerning the use of human patient data, to ensure transparency and replicability of the study.

### 2.7 Systematic review methodologies: strategy, selection criteria, and bias assessment

To ensure methodological rigor and translational relevance, three independent systematic literature reviews (SLRs) were conducted at different research stages: WP2 (focused on endocrine and metabolic dysfunctions in psychiatric disorders), WP3 (on hormonal therapies as adjunctive treatments for mental disorders), and WP5 (on the systemic role of human chorionic gonadotropin in psychiatric care).

Although each review had distinct research questions, eligibility criteria, and databases, they were all developed under a unified methodological framework inspired by PRISMA guidelines and adapted to the Work Breakdown Structure (WBS) model. This approach enabled internal coherence, transparency, and replicability throughout the data collection, screening, and analysis phases.

The following subsections (2.7.1 to 2.7.10) describe the strategies employed across these reviews, including database selection, inclusion and exclusion criteria, data extraction procedures, bias assessment, and synthesis of results. These methodological steps supported the identification of critical success factors and fundamental points of view that inform the central hypothesis of this article.

Our methodology was inspired by the PRISMA guidelines (27), but tailored to align with the WBS framework, ensuring a structured and systematic approach to the literature review process. Although the review protocol was not formally registered, all methodological steps—including search strategy, eligibility criteria, data extraction, and analysis—were carefully documented. Future studies will consider registration to further enhance credibility and transparency.

#### 2.7.1 Information sources and search strategy

A comprehensive search was conducted in the Scopus and Web of Science databases to capture a wide range of relevant studies addressing the key parameters of each WP.

#### 2.7.2 Study selection

Two reviewers independently screened the titles and abstracts of retrieved records. Full texts were assessed for eligibility according to predefined inclusion and exclusion criteria.

#### 2.7.3 Data collection process

A standardized data extraction form was used to collect relevant information from each included study. Extracted data included study characteristics (e.g., author, year of publication), methodologies, and key findings. Two reviewers performed the extraction independently, and discrepancies were resolved through consensus discussions. The extracted data were categorized into fundamental points of view, critical success factors (CSF’s), or indicators, in accordance with the multi-criteria structure of each WP.

#### 2.7.4 Data items

The main data items included definitions, types of biological effects, mechanisms of action, and measurement methodologies. However, each systematic review had a distinct purpose: WP2 focused on mapping fundamental points of view related to endocrine-metabolic dysfunctions in psychiatric disorders; WP3 identified critical success factors (CSF’s) associated with hormonal adjunctive therapies; and WP5 analyzed the integrative potential of hCG as a systemic intervention, aiming at a preliminary validation of the proposed translational hypothesis.

#### 2.7.5 Risk of bias in individual studies

Although standardized tools are not routinely applied to non-clinical systematic reviews, we assessed the methodological quality, measurement validity, and consistency of the evidence across WP2, WP3, and WP5, in alignment with their respective objectives.

#### 2.7.6 Summary measures and synthesis of results

The primary outcome of interest was WP2 and WP3 specific objectives. Results were synthesized narratively, highlighting the variety of biological influence key points recognized, their measurement, and impact.

#### 2.7.7 Additional analyses

Given the heterogeneity of studies, a meta-analysis was not feasible. In response to the PRISMA guideline (27) on "Describe methods used to explore the geometry of the treatment network under study and potential biases related to it," our study employed a comprehensive multi-criteria methodology, in accordance with Bana and Costa et al. (28), to systematically analyze and interpret the complex network of treatments. This approach was instrumental in elucidating the intricate interconnections and potential biases within the compiled evidence base.

#### 2.7.8 Exploration of treatment network geometry

To explore the geometry of the treatment network, we utilized the multi-criteria methodology to construct a visual and analytical representation of the evidence network through tables and figures, subsequently consolidating the presentation of the developed framework.

#### 2.7.9 Identification and mitigation of potential biases

The multi-criteria methodology played a crucial role in identifying and addressing potential biases within the treatment network, particularly by facilitating the exclusion of articles through analytical reading. When a specific FPV, for example, was chosen, the data treatment could be more objective. By systematically evaluating the evidence through these newly predefined criteria, we discerned patterns of bias and implemented corrective measures. Several methodological limitations should be noted. First, the broad inclusion criteria, while allowing for a comprehensive review, may have introduced variability in the quality of the included studies. Second, the exclusion of non-English, non-Portuguese, and non-Spanish studies could result in language bias. Lastly, the lack of a registered review protocol may limit the reproducibility of our SLR. These limitations were mitigated through rigorous quality assessment and consensus discussions among the review team.

#### 2.7.10 Evidence base compilation and description

The final evidence base was presented through a multi-criteria framework, with a clear and accessible synthesis of how each source contributed to the formation of FPV’s and CSF’s. Each included study underwent rigorous quality appraisal, reinforcing the reliability and validity of the dataset. Although a meta-analysis was not performed due to study heterogeneity, the structured narrative synthesis enabled a robust and comprehensive interpretation of the literature relevant to WP2, WP3, and WP5.

In summary, the multi-tiered methodological architecture employed in this study—anchored in PRISMA-guided systematic reviews, the WBS framework, and a multicriteria decision-making approach—enabled the rigorous mapping of conceptual and translational domains relevant to neuroendocrine dysfunction in psychiatric disorders. By operationalizing evidence through the identification of FPVs and their corresponding CSFs, this approach established a structured analytical lens for evaluating hCG’s systemic effects. The synthesis that follows does not merely report empirical findings but reflects the culmination of a strategically aligned research design. It presents a translational scaffold for understanding the physiological mechanisms underlying HHT and its potential relevance as an adjunctive strategy in neuropsychiatric care.

## 3 Results

The results illustrate a complex translational interplay between neuroendocrine crosstalk in the CNS and mitochondria-mediated cellular activity during the course of mental disorders, attempting to adjust human physiology. The integration of data collected in WP2 offers a novel translational perspective on the synergy between various physiological adaptations considered fundamental – defined in this research as fundamental points of view – associated with the described interplay generating a biological impact on the human body, possibly contributing to clinical outcomes.

As a specific result of WP2, it was identified that the stimulation of sex hormone production, reduction of insulin resistance—frequently present in cases of depression and anxiety (34, 35), whether systemic or central (36, 37), as well as associated with leptin resistance (38)—enhancement of hypothalamic activity, increased availability of IGF-1, and potential reduction of mitochondrial dysfunction each lead to various systemic biological benefits. These benefits include improved mood and well-being, decreased systemic inflammation, better regulation of appetite, sleep, and emotion, neuroprotection and cognitive enhancement, and restoration of normal cellular function. Following this, the selection of FPVs was carried out. **Table 2** summarizes the FPVs, their definitions, and the correlated studies, providing a clear and concise overview of the central action points of hormonal replacement adjunctive therapy and its therapeutic implications based on current evidence. All the selected FPVs are highly plausible based on the available literature and are relevant to the specific objectives of WP2. They reflect areas where hormonal and metabolic modulation can have a significant impact on the treatment of psychiatric disorders, supporting the importance of an integrated and systemic approach to these conditions.

**Table 2:** The fundamental points of view selected from the pre-defined parameters by WP2.

FPV	Description & Key References
**Enhancement of Hypothalamic Activity**	The hypothalamus regulates endocrine and autonomic functions, influencing mood, appetite, and sleep. Its modulation can alleviate psychiatric symptoms (8).
**Reduction of Neuroinflammation & Systemic Inflammation**	Insulin sensitivity improvements reduce inflammation, a key factor in psychiatric disorders such as depression and schizophrenia (11, 39).
**Brain-Gut-Microbiota Axis Involvement**	The gut microbiota influences neurotransmitter production, inflammation, and epigenetic modulation, impacting psychiatric conditions (40–42).
**Stimulation of Sex Hormone Production**	Testosterone (in men) and estradiol (in women) modulate mood, energy, and neurotrophic factors, contributing to psychiatric symptom relief (43).
**Increased IGF-1 Availability**	IGF-1 promotes neurogenesis and cognitive function, offering neuroprotection in psychiatric and neurodegenerative disorders (44, 45).
**Potential Reduction of Mitochondrial Dysfunction**	Mitochondrial impairment is linked to psychiatric disorders; metabolic and hormonal interventions may restore function (46, 47).

As a result of WP3, it is notable how hormone replacement therapy is still not a focus of research as an adjunct treatment for mental disorders. There is substantial evidence linking mood to hormone replacement therapy, but adjunct strategies in psychiatric treatment are essentially limited to two options: transdermal estradiol therapy in postmenopausal women, associated with progesterone replacement when they have a uterus –considered a second-line treatment with level 2 evidence (48), and DHEA supplementation, considered a third-line treatment (13). Interestingly, it is clear in the guidelines that one of the main focuses of maintenance treatment is the reestablishment of performance and quality of life to pre-morbid levels, with encouragement to request laboratory tests for the evaluation of endocrine dysfunctions, such as TSH levels (13). However, it remains evident that these treatments are often viewed as addressing comorbidities rather than primary psychiatric conditions. There is still no widespread consensus or understanding that these symptoms may be manifestations of a systemic disease involving endocrinological dysfunctions in the central nervous system, which are associated with mitochondrial dysfunction and subsequently cause an imbalance between excitatory and inhibitory neurotransmitters. Mitochondrial dysfunction plays a critical role in the pathogenesis of depression by contributing to synaptic impairment, neuroinflammation, and energy depletion. The accumulation of defective mitochondria accelerates neuronal dysfunction, exacerbating depressive symptoms. (46, 47) and probably doing the same in other mental disorders (49, 50). The symptoms are understood as stemming from distinct diseases; hence, treatments are not scientifically explored as adjuncts.

In the past, there was a particular moment when an attempt was made to consider the process as a single entity. Research such as that by Nerozzi et al. (51) and Schatzberg and Nemeroff (52) tried to explore the unified concept. These studies were part of broader efforts to understand the intricate relationships between hypothalamic function and psychiatric disorders. Each book related to this research contains papers presented at symposia focusing on the relationship between hypothalamic function and psychiatric disorders. The first symposium, largely American and held in 1985, concentrated on the neuropeptide corticotropin-releasing hormone (CRH) and its potential role in the pathophysiology and pathogenesis of depressive illness. This event highlighted the importance of CRH in the stress response and its implications for mental health. The second symposium, international in scope and held in 1986, expanded the focus to include a wider range of neuropeptides—such as vasopressin, oxytocin, angiotensin, somatostatin, and opioids—in addition to CRH. It also covered a broader spectrum of clinical disorders, including anorexia nervosa, dementia, epilepsy, and erectile impotence, alongside depression. This broader approach aimed to provide a more comprehensive understanding of how different neuropeptides influence various psychiatric and neurological conditions. This likely reflects the overlapping research themes and the continuity of their work across different symposia. By exploring these unified concepts, researchers like by Nerozzi et al. (51) and Schatzberg and Nemeroff (52) contributed to a foundational understanding of the neuroendocrine mechanisms underlying psychiatric disorders. Their work underscores the importance of considering the interconnectedness of hormonal, neurochemical, and physiological processes in developing comprehensive treatment approaches. This historical context highlights the relevance of integrated models provided by a WBS methodology in current research. For instance, the specific objective of WP3 was to generate an updated theoretical reference on the use of hormonal therapies to address endocrine and metabolic dysfunctions in psychiatric disorders.

The actions of WP4 involved the integration of data from WP2 and WP3 to enable a reinterpretation through translational research to apply Design Thinking and Open Innovation for innovative solutions in medicine. This exploration led to the development of numerous hypotheses that could potentially enhance the benefits of current psychiatric treatments by incorporating the identified knowledge. To achieve this, we sought to identify a stimulus that could unify an approach addressing the FPVs identified in WP2 and continue offering similar benefits to the treatments identified in WP3. The authors sought a common denominator for their reflections. The hormone Human Chorionic Gonadotropin (hCG) is a great endocrine stimulant associated with life development (53), whose influence is fundamental for all the bodily adaptations associated with pregnancy (54–56). From this point onwards, the focus shifted towards understanding the impact of each FPV through a hormetic dose of hCG, resulting in corresponding effects on cellular, tissue, or bodily adaptations that could enhance psychiatric treatments: an hCG Hormetic Therapy (HHT). Thus, in a translational reinterpretation, hCG can stimulate neuroendocrine crosstalk—and the probable alterations in body metabolism induced by these stimuli can help patients dealing with mental disorders—formulating the novel medical hypothesis.

Human chorionic gonadotropin (hCG) is a multifaceted hormone with critical roles in reproductive biology and beyond. Produced by different cell types, hCG and its variants exhibit a range of biological functions essential for pregnancy and overall health. According to the National Institute of Diabetes and Digestive and Kidney Diseases (57), hCG is involved not only in the maintenance of pregnancy but also has diverse functions that extend to overall health and cellular adaptation. This glycoprotein promotes progesterone production by corpus luteal cells, which is vital for maintaining the early stages of pregnancy (58). It also enhances angiogenesis in the uterine vasculature, ensuring an adequate blood supply to the developing placenta and fetus (54). Additionally, it has anti-inflammatory properties, as it suppresses macrophage activity and immune responses against the fetus, protecting the developing embryo from maternal immune rejection (59). Its hyperglycosylated form promotes cytotrophoblast cell invasion during implantation and has been linked to the growth and malignancy of choriocarcinoma cells (60). Collectively, these diverse actions underscore hCG’s pivotal role in reproductive and systemic health, highlighting its potential therapeutic applications (53).

The relevance of humoral signaling pathways in communicating the body’s physiological state to the brain has been highlighted in recent literature (61). These pathways, which include humoral and cellular signals, can disrupt neuronal structure, chemistry, and function, leading to discrete behavioral changes. In the context of infection and inflammation, these pathways play a critical role in the pathophysiology of psychiatric disorders. Integrating these findings, we propose that hCG, through its immunomodulatory and humoral properties, can positively influence neuroendocrine crosstalk and thus offer a new therapeutic perspective for treating psychiatric disorders as systemic conditions.

Considering the neuroendocrinological interaction stimulated by hCG, the application of a multi-criteria methodology in WP5 allows for the systematic classification and evaluation of these factors, ensuring that the therapeutic strategies developed are based on robust evidence and a thorough understanding of the underlying biological mechanisms. Reinterpreting the FPVs identified by WP2 to assess the potential therapeutic action of hCG was a key action of WP5. The review of WP2 FPVs in WP5 is presented in **Table 3**.

**Table 3:** Potential actions of hCG on key neuroendocrine pathways.

FPV	Potential hCG Action	Key References
Enhancement of Hypothalamic Activity	hCG modulates hypothalamic activity, improving appetite, sleep, and emotional regulation, which may positively impact psychiatric disorders.	Li et al. (8)Bayram et al. (62); Visentin et al. (63); Hershko Klement and Shulman (64)Adibi et al. (65); Rivero-Müller and Huhtaniemi (66); Borisova et al. (67)
Reduction of Neuroinflammation & Systemic Inflammation	hCG’s immunomodulatory effects reduce systemic and neuroinflammation, promoting mood stability and lowering anxiety.	Alam et al. (40); Chaudhary et al. (68); Dubois et al. (69); Mukherjee et al. (70)Paulesu et al. (71); Rao (72, 73); Schumacher and Zenclussen (74) Huang et al. (75) Adibi et al. (65) Gridelet et al. (76) Gallardo et al. (59) Moldogazieva, et al. (77)
Brain-Gut-Microbiota Axis Involvement	hCG modulates gut microbiota and inflammatory responses, influencing psychiatric symptom relief via the gut-brain axis.	Alam et al. (40) Churchward et al. (78) Schüler-Toprak et al. (79) Gridelet et al. (76)
Stimulation of Sex Hormone Production	hCG stimulates Leydig (testes) and theca/granulosa cells (ovaries), increasing testosterone and estrogen levels, improving mood and cognition.	Fournier (55)Nwabuobi et al. (56)Casarini et al. (80); Misra & Mohanty (81); Rivero-Müller and Huhtaniemi (66) Schüler-Toprak et al. (79) Fournier et al., (54) Churchward et al. (78)
Increased IGF-1 Availability	hCG influences the HPA axis and hormonal modulation, potentially elevating IGF-1 levels, which may protect against neuropsychiatric decline.	Shah et al. (82); Peixoto et al. (83); Nwabuobi et al. (56)Stenman (84)
Potential Reduction of Mitochondrial Dysfunction	hCG enhances mitochondrial biogenesis and function, mitigating psychiatric symptoms associated with cellular energy deficits.	Miller et al. (85); Hill & Spencer-Segal (86); Gallardo et al. (59) Zhu et al. (87) Smitz and Platteau (88) Rao (89)

Additionally, the application of the Multi-Criteria Methodology in WP5 classified the CSF’s based on their approach similarities concerning the FPV’s, in accordance with Yew Wong and Aspinwall (90) and Venkataraman and Cheng (91). The FPV’s highlight the key mechanisms through which hCG can influence neuroendocrine and immune pathways. These mechanisms form the basis for identifying critical success factors (CSF’s) that are directly related to the therapeutic potential of hCG in psychiatric disorders. The CSF’s are presented as the fundamental elements of the biological action of hCG, based on a translational reinterpretation derived from integrating the FPV’s, as previously defined (**Table 4**).

**Table 4:** Critical Success Factors (CSFs) of hCG with potential psychiatric implications.

CSF	Description	Key References
Testosterone Modulation	hCG binds to LH receptors on Leydig cells, increasing testosterone production, which enhances mood and well-being.	Mikhalitskaya et al. (92); Nwabuobi et al. (56) Santen et al. (93)Lin et al. (94); Mizrachi et al. (95); Alexander et al. (96) Barbonetti et al. (97) Boeri et al. (98)
Estrogen Modulation	hCG activates LH/hCG receptors on ovarian cells, increasing estrogen production, which enhances mood and cognitive function.	Nwabuobi et al. (56) Churchward et al. (78) Schüler-Toprak et al. (79) Casarini et al. (80); Misra & Mohanty (81) Ycaza Herrera et al. (99) Xu et al. (100) Sisinni & Landriscina (60)
Progesterone Stimulation	hCG stimulates progesterone production, which has calming effects on the brain, promoting mood stability and reducing anxiety.	Andersen et al. (101) Berger and Lapthorn (102) Moldogazieva et al. (77) Querat (103)
DHEA Production	hCG increases DHEA levels, which are linked to improvements in depression, particularly in treatment-resistant cases.	Shah et al. (82) Nenezic et al. (104) Peixoto et al. (83)
Insulin Sensitivity & Anti-inflammatory Effects	hCG improves insulin sensitivity and reduces systemic inflammation, contributing to metabolic and mood stability.	Hill et al. (105); Bayram et al. (62) Casarini et al. (80) Sisinni & Landriscina (60) Wang et al. (34) Mukherjee et al. (70) Furcron et al. (106) Gridelet et al. (76) Khan & Benner (107)
Hypothalamic Regulation	hCG enhances hypothalamic function, positively impacting appetite, sleep, and emotional regulation.	Visentin et al. (63); Hershko Klement and Shulman (64)Forbes (108)
Mitochondrial Function	hCG may enhance mitochondrial biogenesis, playing a role in mitigating psychiatric symptoms linked to energy metabolism deficits.	Sisinni and Landriscina (60)Casarini et al. (80) Querat (103)
Neuroplasticity & Synaptic Plasticity	hCG may support synaptic remodeling and cognitive functions, potentially reducing neurodegenerative risks.	Pei and Wallace (7) Rao (73) Li et al. (109) Paternina-Die et al. (110) Wang et al. (111); Meng et al. (112); Wan et al. (113)
LH-like Action	hCG mimics LH activity, regulating steroidogenesis and potentially improving cognitive function.	Hershko Klement and Shulman (64)Lazzaretti et al. (114) Litwicka et al. (115) Alsbjerg et al. (116)
Cortisol Regulation	hCG influences glucocorticoid receptor signaling, modulating the stress response and potentially improving psychiatric symptoms.	Rao (117); Jeon et al. (118)
Thyroid Hormone Modulation	hCG may affect thyroid hormone levels via cross-reactivity with TSH receptors, influencing metabolic and mood regulation.	Nwabuobi et al. (56) Paulesu et al. (71) Kennedy and Darne (119) Chivukula et al. (120) Lamos & Munir (121)

By focusing on these identified FPV’s and CSF’s, we can better understand the multi-faceted roles of hCG in modulating neuroendocrine function, immune responses, and hormonal balance. This approach allows for the development of targeted interventions that leverage the specific physiological actions of hCG, ultimately enhancing the efficacy of psychiatric treatments. Additionally, the integration of FPV’s and CSF’s provides a comprehensive framework for understanding and optimizing HHT. This comprehensive analysis paves the way for innovative therapeutic approaches that can significantly improve patient outcomes in the context of psychiatric disorders.

The concept of hormesis refers to a biphasic dose-response relationship where low doses of a substance can stimulate beneficial effects, while higher doses may be detrimental (122, 123). Applying this principle, the weekly administration of hCG at low, hormetic doses is proposed as a novel adjunctive strategy in psychiatric treatment. This approach leverages the multiple modulatory actions of hCG on the HPA axis, its immunomodulatory properties, and its ability to influence hormonal balance without overwhelming the body’s physiological systems. By harnessing the hormetic effects, the aim is to optimize the therapeutic benefits of hCG, enhancing neuroendocrine crosstalk and potentially improving mood, cognitive function, and overall mental health outcomes. This strategy is grounded in emerging evidence suggesting that carefully controlled, low-dose interventions can elicit adaptive responses that promote resilience and recovery in neuropsychiatric disorders. Additionally, patient selection is critical to ensure safety and efficacy. Patients should be thoroughly screened for contraindications, such as hormone-sensitive conditions or thromboembolic disorders. To mitigate potential thrombotic risks associated with HHT, the concurrent administration of 81 mg of acetylsalicylic acid daily is recommended. Furthermore, a treatment duration of 10 to 20 weeks with intermittent rest periods is suggested to potentiate physiological adaptation. Importantly, this current research has identified no potential risk of drug interactions with existing pharmacological treatments, allowing hCG to be added at any point deemed appropriate by the clinician or researcher, following the adequate selection of patients or research participants. This combined approach aims to balance the therapeutic benefits of hCG with the highest standards of health safety.

A SMART and FINER analysis was carried out in WP6 to assess the potential impact of this novel medical hypothesis. It was scrutinized against the goals of being specific, measurable, achievable, relevant to neuropsychiatric treatments, and having a defined timeline for accomplishment (**Table 5**).

**Table 5:** SMART and FINER analysis.

Criteria	Analysis
SMART	
**Specific**	The study focuses on hCG as a hormetic adjunctive treatment for psychiatric disorders, examining its effects on the neuroendocrine axis, mitochondrial function, and inflammation.
**Measurable**	Outcomes are evaluated through hormone levels (e.g., IGF-1, testosterone, estradiol), reductions in neuroendocrine and systemic inflammation, mitochondrial function biomarkers, and psychiatric symptom assessments.
**Achievable**	The administration of hormetic doses of hCG is clinically feasible within current safety standards, with acetylsalicylic acid co-administration to mitigate thrombotic risks.
**Relevant**	This approach integrates endocrinology and psychiatry, offering a novel treatment strategy with potential benefits for patients with mental disorders.
**Time-bound**	The proposed treatment duration of 10 to 20 weeks, with planned rest intervals, provides a structured timeline for evaluating therapeutic effects and adaptive responses.
FINER	
**Feasible**	The study is practical within current clinical settings, leveraging known safe doses of hCG and ensuring patient safety through careful selection and continuous monitoring.
**Interesting**	The hypothesis integrates hormesis and hormonal therapy in psychiatry, contributing to a novel and intriguing avenue of research.
**Novel**	The application of hCG in hormetic doses for psychiatric disorders represents an innovative therapeutic perspective beyond its conventional use.
**Ethical**	Ethical considerations include rigorous patient selection, continuous monitoring, and risk mitigation through acetylsalicylic acid co-administration.
**Relevant**	Findings may redefine psychiatric treatment paradigms by introducing hormonal modulation as a systemic approach to mental health.

The use of hCG in hormetic doses, as analyzed under the SMART and FINER criteria, offers an innovative and well-founded approach to treating psychiatric disorders. This methodology ensures the feasibility and relevance of the hypothesis, paving the way for new therapeutic strategies that can significantly improve clinical outcomes in patients with mental disorders.

## 4 Discussion

Sachar (124) delineated the relationship between neuroendocrine dysfunctions and mental illnesses such as depression. This study highlights hormonal abnormalities in patients with severe depression, including hypersecretion of cortisol, resistance to dexamethasone suppression, diminished HGH (human growth hormone) responses to insulin-induced hypoglycemia, and decreased TSH (thyroid-stimulating hormone) responses to TRH (thyrotropin-releasing hormone). Furthermore, it reports relatively decreased plasma concentrations of LH (luteinizing hormone) in postmenopausal women with primary unipolar depression. These findings support the hypothesis that hypothalamic dysfunction may be implicated in certain forms of depressive illness, consistent with contemporary theories about abnormal monoamine activity in the brain. Additionally, research presented by Nerozzi et al. (51) and Schatzberg and Nemeroff (52) reinforced the importance of neuroendocrine crosstalk in neuropsychiatric disorders, suggesting that hormonal regulation plays a fundamental role in the pathophysiology of mental illnesses.

Building on this foundation, this study advances the understanding of neuroendocrine dysfunctions in psychiatric disorders by exploring the potential of HHT as a therapeutic approach. By integrating historical data with contemporary research, it is possible to outline a framework in which neuroendocrine interactions are leveraged for novel treatment strategies. Sachar’s early contributions underscored the importance of hormonal responses in mental illnesses as a window into altered brain function. His anticipation of advancements in radioimmunoassay techniques reflects an expectation that further elucidation of neuroendocrine pathways would refine both diagnostic and therapeutic strategies. Recent developments in neuroscience, endocrinology, and psychiatry have provided a more comprehensive perspective on these interactions, reinforcing the need to revisit neuroendocrine mechanisms as a basis for improving clinical outcomes. The translational reinterpretation proposed in this study aligns with this broader perspective, emphasizing the potential of HHT to address key gaps in psychiatric treatment.

The implications of hypothalamic dysfunction extend beyond depression and encompass a range of neurological and psychiatric conditions, including dementia and eating disorders. Gottfries et al. (125) and Balldin et al. (126) have explored the role of hypothalamic dysfunction in dementia, highlighting how alterations in this critical brain region contribute to cognitive decline. The hypothalamus regulates not only endocrine functions but also appetite, sleep, and circadian rhythms, all of which can be disrupted in neurodegenerative disorders. Similarly, hypothalamic dysfunction has been implicated in eating disorders, particularly in cases of binge eating, where dysregulation of appetite-related hormones such as leptin and ghrelin may drive pathological feeding behaviors. This research underscores the potential of therapeutic strategies aimed at modulating hypothalamic function to mitigate these effects and restore homeostasis.

The integration of neuroendocrine mechanisms into psychiatric treatment approaches is a necessary evolution in the field. Treatments aimed at restoring or modulating hypothalamic function may offer new hope for patients affected by complex neuropsychiatric conditions. Moreover, hCG’s pleiotropic effects (76), including its role in stimulating sex hormone production, reducing insulin resistance, improving hypothalamic function, increasing IGF-1 availability, and potentially alleviating mitochondrial dysfunction—as also evidenced by its ability to protect dopaminergic neurons from cell death in Parkinson’s disease models (127)—suggest a broad spectrum of therapeutic possibilities. The findings of this study indicate that HHT could exert beneficial effects in psychiatric disorders by modulating endocrine and metabolic pathways, mitigating neuroinflammation, and supporting neuronal and mitochondrial function. However, the complexity of these interactions necessitates further targeted investigation to fully explore the clinical potential of hCG in psychiatric contexts.

Additionally, by reinforcing hormonal and metabolic balance while modulating neuroendocrine function, the proposed HHT may also foster greater treatment adherence and overall well-being. In this regard, recent evidence from a meta-analysis of Self-Determination Theory-informed health interventions indicates that strategies promoting autonomy, competence, and relatedness are strongly associated with improved psychological and physical health outcomes, further validating the relevance of integrative and patient-centered approaches in psychiatric care (128). While further investigation into the therapeutic applications of hCG in psychiatric treatment is essential, it is equally important to consider the contentious debates and safety concerns surrounding its use in other contexts, such as the hCG diet (129). The scientific controversy surrounding this diet approach is marked by conflicting reports on its efficacy and safety, raising significant concerns among medical professionals. Goodbar et al. (130) detailed a case of a 64-year-old woman who developed deep vein thrombosis (DVT) and bilateral pulmonary embolisms shortly after starting the HCG diet. Despite an extensive workup revealing no other risk factors for thrombosis, the patient's adverse events were attributed to the diet, highlighting the potential dangers of this weight loss strategy. The study emphasized that while the hCG diet has garnered popularity, it lacks robust efficacy and safety data, with only one out of six relevant studies showing significant weight loss, all based on the controversial Simeons method involving hormone injections and severe calorie restriction. Additionally, the marketed forms of hCG, including sublingual drops and lozenges, lack evidence-based standards for safety and efficacy. This case highlights the importance of the hormetic dose concept and the appropriate consideration of adding a small dose of acetylsalicylic acid to the suggested clinical protocol. Butler and Cole (131) further reinforced these concerns by reviewing the literature and presenting supporting data.

The exploration of hCG as a hormetic adjunctive treatment is supported by its capacity to influence key neuroendocrine and metabolic pathways implicated in psychiatric disorders. Martin and Riskind (132) highlight the profound impact of hypothalamic dysfunction on appetite, sleep, and emotional regulation, further reinforcing the need for interventions that address these interconnected systems. The clinical descriptions of hypothalamic dysfunction cases emphasize the wide-ranging effects of lesions in this region, including disruptions in energy homeostasis, water balance, circadian regulation, and behavior. Given these multifaceted roles, interventions that enhance hypothalamic function may provide a novel approach to psychiatric treatment.

**Figure 6:**
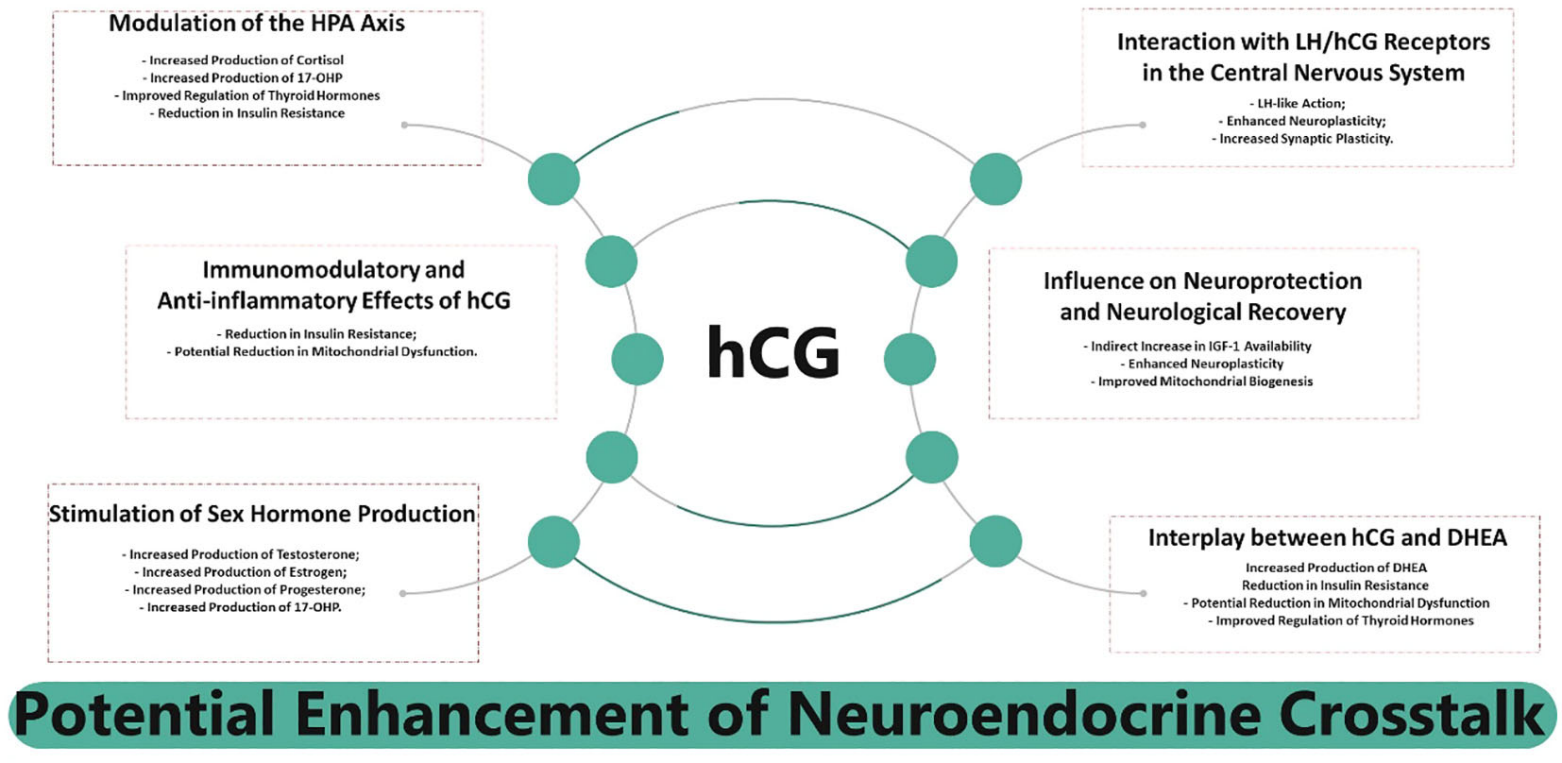
Summarized interplay between human Chorionic Gonadotropin (hCG) and neuroendocrine crosstalk.

Treatments aimed at restoring or modulating hypothalamic function may offer new hope for patients affected by these conditions. The clinical relevance of these neuroendocrine interactions underscores the importance of identifying potential therapeutic targets. In this context, the exploration of hCG as a hormetic adjunctive treatment is supported by its capacity to modulate key endocrine and metabolic pathways implicated in psychiatric disorders. The HHT, by influencing hormone production, insulin sensitivity, IGF-1 availability, and potentially mitochondrial function, may offer a multifaceted approach to mitigating some of the negative impacts of hypothalamic dysfunction. This provides valuable insights into the neurological manifestations of hypothalamic diseases, highlighting the central importance of the hypothalamus in regulating a wide range of bodily and behavioral functions. This review identifies several areas of potential intersection, as detailed in **Table 6**, and summarizes these interactions in **Figure 6**.

**Table 6:** Potential Intersections of – hCG Hormetic Therapy (HHT) with Hypothalamic Dysfunction and Psychiatric Disorders.

Area	Potential Intersection with HHT
**Hormonal and Metabolic Regulation**	The hypothalamus plays a central role in maintaining hormonal and metabolic homeostasis (133). Dysfunction in this region is linked to endocrine, metabolic, and psychiatric disorders (134–136), as observed in documented cases (137). HHT, by modulating sex hormone production and potentially influencing broader endocrine functions, may aid in restoring homeostatic balance.
**Impact on Behavior and Emotion**	Hypothalamic dysfunction is associated with behavioral disturbances, including hyperphagia (138, 139), depression (140), dementia (125), and irritability (132). Given its effects on the hypothalamic-pituitary-gonadal axis and insulin sensitivity, HHT may contribute to mood stabilization and behavioral regulation (136).
**Cognitive Function and Sleep Regulation**	Cognitive impairment, memory deficits, and sleep disturbances are frequently observed in hypothalamic dysfunctions (141). By improving metabolic efficiency and increasing IGF-1 availability, HHT may enhance cognitive performance and sleep quality through direct hypothalamic modulation and systemic neuroprotective effects.
**Management of Physical and Metabolic Symptoms**	Hypothalamic dysfunction can manifest as dysregulated water balance (e.g., polydipsia) and metabolic disturbances (142). Through its endocrine and metabolic effects, HHT may help alleviate these symptoms, indirectly benefiting overall mental health.

Expanding on these findings, this study also examines the clinical application of hCG, with particular attention to dosing regimen, safety profile, and integration into existing treatment frameworks. The proposed dosing regimen for hCG as a hormetic adjunctive treatment for psychiatric disorders was developed based on established clinical protocols, ensuring an appropriate balance between efficacy and safety. Given hCG’s well-documented biological effects and its prior therapeutic applications—including reproductive medicine, metabolic regulation, and neuroendocrine modulation, as demonstrated in systematic reviews on ovarian stimulation protocols and freeze-all in vitro fertilization (IVF) cycles (143)—its use in hormetic doses aligns with established clinical practices. The rationale for this dosage follows the principles of hormesis, a biphasic dose-response phenomenon in which low doses elicit adaptive and beneficial effects, whereas higher doses may lead to adverse outcomes. The selection of dose and treatment duration was guided by empirical data from prior clinical studies, which demonstrated the safety of hCG when administered over extended periods within controlled dose ranges. Notably, long-term therapeutic applications of hCG have not been associated with significant adverse events when maintained within appropriate physiological limits.

The decision to establish 500 IU as the baseline dose in future studies is supported by robust clinical evidence. A recent meta-analysis by Luo et al. (144) evaluated intrauterine hCG perfusion in recurrent implantation failure (RIF) and identified 500 IU as the most effective dose in improving clinical pregnancy and embryo implantation rates without significantly increasing miscarriage rates. This meta-analysis, which synthesized data from 13 studies including 2,157 participants, demonstrated a clear advantage of the 500 IU dosage across multiple clinical outcomes. Importantly, subgroup analyses indicated that while this dose provided consistent benefits, higher doses (1000 IU and 2000 IU) produced conflicting results and raised concerns regarding potential side effects. Given these uncertainties, the 500 IU dosage emerges as the most reliable starting point for future investigations into its potential psychiatric applications. Furthermore, this dosage is applicable to both men and women, as hCG plays a crucial role in modulating gonadal steroidogenesis and neuroendocrine function in both sexes, reinforcing its translational potential beyond reproductive contexts. Evidence from previous studies, such as Amory et al. (145), demonstrates that hCG administration in men effectively stimulates intratesticular testosterone production in a dose-dependent manner, further supporting the safety and endocrine relevance of the 500 IU regimen in male populations.

Additionally, the lack of long-term follow-up data in current literature underscores the necessity of further research to assess potential delayed side effects. This consideration justifies the proposed treatment duration of 10 to 20 weeks, allowing for controlled evaluation of its physiological impact while minimizing the risk of cumulative adverse effects. Although hCG is generally well tolerated, potential side effects must be carefully monitored. Reported adverse effects in other therapeutic applications include mild fluid retention, transient headaches, and localized injection site reactions. In rare cases, thromboembolic events have been observed, particularly in predisposed individuals. To mitigate such risks, the concurrent administration of low-dose acetylsalicylic acid has been proposed, given its established role in reducing thrombotic complications in similar clinical contexts.

The integration of hCG into psychiatric treatment protocols requires a multidisciplinary approach. Given its potential influence on endocrine and metabolic regulation, neuroinflammation, and mitochondrial function, hCG could serve as an adjunctive therapy alongside conventional psychotropic medications and lifestyle interventions. Individualized patient assessment, close monitoring of metabolic parameters, and consideration of potential synergistic effects with existing therapies will be crucial for successful implementation. This framework ensures that the proposed intervention remains within the scope of evidence-based practice, while acknowledging its exploratory nature in psychiatric disorders. Future clinical investigations will be essential to refine dosing parameters, establish long-term safety profiles, and optimize its role within personalized treatment strategies. Additionally, controlled trials evaluating different dosing regimens and their impact on endocrine, metabolic, and neuropsychiatric parameters will be critical for validating its clinical application beyond reproductive medicine.

While this study provides compelling evidence for the potential benefits of a hormetic dose of hCG in psychiatric treatment, further clinical trials are required to validate its safety and efficacy in specific patient populations. Large-scale studies will be necessary to fully elucidate its neuroendocrine effects and establish standardized treatment protocols. This research contributes to the growing body of evidence supporting the role of neuroendocrine modulation in psychiatric treatment and highlights the need for ongoing investigation into the therapeutic applications of hCG within a precision medicine framework.

## 5 Conclusions

By integrating data from multiple work packages (WPs), this research has identified key functional points of view (FPVs) and critical success factors (CSF’s) that highlight the multifaceted roles of hCG in enhancing hypothalamic activity, reducing neuroinflammation and systemic inflammation, influencing the brain-gut-microbiota axis, stimulating sex hormone production, increasing the availability of IGF-1, and potentially reducing mitochondrial dysfunction.

The application of hCG in a hormetic dose framework offers a novel approach to psychiatric treatment, leveraging its systemic effects to promote hormonal balance, improve metabolic function, and support neuronal health. The inclusion of acetylsalicylic acid to mitigate thrombotic risks, along with a structured treatment duration of 10 to 20 weeks with intermittent rest periods, underscores a commitment to maximizing therapeutic benefits while maintaining patient safety.

Importantly, this current research has identified no potential risk of drug interactions with existing pharmacological treatments, allowing for flexible integration into clinical practice. The proposed HHT can be added at any point deemed appropriate by clinicians, following careful patient selection to ensure suitability and efficacy. The use of the Work Breakdown Structure (WBS) methodology has ensured a systematic and structured approach to research, enhancing the robustness of findings and their applicability to clinical practice.

While this study provides a strong theoretical framework, empirical validation through clinical trials is crucial. Future research should prioritize controlled trials to refine dosing regimens, assess long-term safety, and optimize its integration within psychiatric care. Additionally, gender-specific responses to hCG administration warrant further investigation to ensure equitable clinical applicability.

The ethical considerations surrounding the off-label use of hCG in psychiatric disorders are an important aspect of this discussion. Future studies must adhere to rigorous ethical guidelines, including independent review board (IRB) approval, adherence to Good Clinical Practice (GCP) standards, and transparent data reporting to establish a comprehensive risk-benefit profile. Given its potential endocrine and metabolic effects, long-term monitoring and follow-up studies will be essential in ensuring patient safety.

In conclusion, this research provides a translational foundation for the application of hCG in psychiatric disorders, opening new avenues for innovative, personalized, and effective therapeutic strategies. The interdisciplinary approach, combining insights from neuroscience, endocrinology, and psychiatry, is pivotal for advancing treatment paradigms and improving patient outcomes.

## Data Availability

The raw data supporting the conclusions of this article will be made available by the authors, without undue reservation.
